# Mitochondrial Nucleoid: Shield and Switch of the Mitochondrial Genome

**DOI:** 10.1155/2017/8060949

**Published:** 2017-06-07

**Authors:** Sung Ryul Lee, Jin Han

**Affiliations:** ^1^Department of Integrated Biomedical Science, Cardiovascular and Metabolic Disease Center, College of Medicine, Inje University, Busan, Republic of Korea; ^2^National Research Laboratory for Mitochondrial Signaling, Department of Physiology, Cardiovascular and Metabolic Disease Center, College of Medicine, Inje University, Busan, Republic of Korea

## Abstract

Mitochondria preserve very complex and distinctively unique machinery to maintain and express the content of mitochondrial DNA (mtDNA). Similar to chromosomes, mtDNA is packaged into discrete mtDNA-protein complexes referred to as a nucleoid. In addition to its role as a mtDNA shield, over 50 nucleoid-associated proteins play roles in mtDNA maintenance and gene expression through either temporary or permanent association with mtDNA or other nucleoid-associated proteins. The number of mtDNA(s) contained within a single nucleoid is a fundamental question but remains a somewhat controversial issue. Disturbance in nucleoid components and mutations in mtDNA were identified as significant in various diseases, including carcinogenesis. Significant interest in the nucleoid structure and its regulation has been stimulated in relation to mitochondrial diseases, which encompass diseases in multicellular organisms and are associated with accumulation of numerous mutations in mtDNA. In this review, mitochondrial nucleoid structure, nucleoid-associated proteins, and their regulatory roles in mitochondrial metabolism are briefly addressed to provide an overview of the emerging research field involving mitochondrial biology.

## 1. Introduction

**Figure 1:**
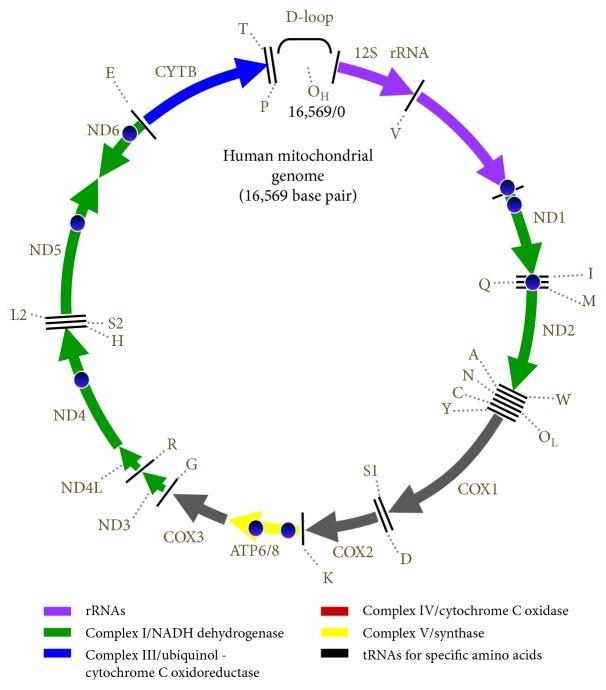
Human mtDNA. Human mtDNA is circular, with 16,569 bps that encode seven of the 43 subunits of complex I, one of the 11 subunits of complex III (CYTB), three of the 13 subunits of complex IV (COXI, COXII, and COXIII), and two of the 16 subunits of complex V (ATP synthase 6 and ATP synthase 8). It also encodes two ribosomal RNAs and 22 transfer RNAs. ATP, adenosine triphosphate; COX, cyclooxygenase; CYTB, cytochrome B; mtDNA, mitochondrial DNA.

Normal cellular physiology is critically dependent upon energy in eukaryotic cells, making mitochondria indispensable organelles for energy production in the form of adenosine triphosphate (ATP) via the electron-transport chain and oxidative phosphorylation system (OXPHOS). Additionally, numerous biological functions, including ATP transport, heat production, metal homeostasis, and stress signaling and defense responses, involve mitochondria [1–5]. Stationary (or immobilized) mitochondria serve as calcium buffers to avoid harmful intracellular calcium overload. Depending on cellular demand, their composition is highly variable from tissue to tissue to enable fulfillment of specialized functions, with accumulation at regions of high-energy demand [4, 6]. The position of mitochondria within the cell is determined largely by the cytoskeleton, which comprises a highly dynamic network of actin filaments, microtubules, and intermediate filaments [7, 8]. Mitochondrial movement, which appears to be influenced by intermediate filament proteins, is highly coordinated with changes in organelle shape in order to produce mitochondria with sizes compatible with their movement [9]. Therefore, the correct distribution of mitochondria is achieved by directed movement and docking and anchoring mechanisms [8]. Unlike other subcellular organelles, such as Golgi, lysosomes, and endosomes, mitochondria individually encapsulate their own genome, referred to as mitochondrial DNA (mtDNA). The size range of mtDNAs found in multicellular animals is relatively narrow (~16.5 kb; Figure 1), with some exceptions varying from 14 kb in the nematode to 42 kb in the scallop [10]. However, the mitochondrial genome of higher plants is much larger than that in multicellular animals, ranging from 200 kb to 2400 kb [6, 10]. Many aspects of mtDNA differ from those of nuclear DNA, including its non-Mendelian genetics and the polyploid nature of the genome within a single cell [11, 12].

Mitochondria preserve very complex and unique machinery to maintain and express the content of mtDNA. For example, mtDNA replication occurs independent of the cell cycle and irrespective of the replication of genes in the nucleus [13]. Mutations originating from chromosomal DNA cannot completely explain mitochondrial diseases manifested in cardiomyopathies [14, 15], neurodegenerative diseases, aging [16–18], and cancer. Mitochondrial genomes are not naked but rather packaged into chromosome-like organellar nuclei, termed nucleoids, that exhibit a discrete macromolecular assembly that dictates mtDNA-protein interactions related to mitochondrial genetics [19]. In eukaryotic cells, thousands of mtDNA molecules are organized into several hundred nucleoids [1, 13, 19–24], which function as units of mtDNA propagation for mtDNA replication, segregation, and gene expression [25–28]. As an organizing body of mtDNA, nucleoids work as a platform for the subtle and controlled regulation of mitochondrial genomes and their efficient integration into cellular signaling [26, 29]. Naked mtDNA in the mitochondrial matrix would preclude efficient mtDNA maintenance, resulting in increased accumulation of mutations and the inevitable faulty segregation of mtDNA. Numerous cellular metabolic processes are connected to dynamic regulation associated with mitochondrial nucleoids in order to control the stabilization, maintenance, distribution, and inheritance of the mitochondrial genome [30, 31]. In this review, we addressed the putative mitochondrial nucleoid structure, proteins involved in nucleoid formation, and their regulatory roles in mitochondrial metabolism. Although in-depth mechanistic findings regarding mtDNA nucleoids have been extensively revealed in model organisms, such as *Saccharomyces cerevisiae* [32], this review will be limited to findings from human and mammalian systems.

## 2. Mitochondrial Structure and Shape

**Figure 2:**
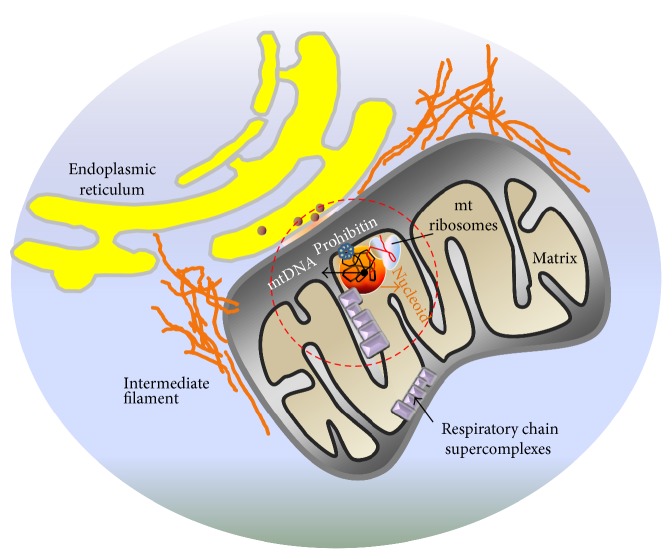
Spatial organization (localization) of mitochondrion and mitochondrial nucleoids. The contour length of circular mtDNA (~16.5 kb) is ~5 *μ*m [47] and requires tight packaging into nucleoids to fit into the tubules of the mitochondrial network comprised of cylinders ~250 nm to ~400 nm in diameter. Nucleoids are organized in higher-ordered assemblies, including respiratory chain supercomplexes [31] and ER-mitochondria complexes (red circle). ER, endoplasmic reticulum; mtDNA, mitochondrial DNA.

Mitochondrial morphology, suggested as ovoid or rod-shaped, is not static, and mitochondria have no fixed size but vary in appearance of the cristae and structure, which can be branched, curved, or elongated and rod-like and fragmented into multiple smaller mitochondria depending on cell type [33–35]. Even within individual cells, mitochondrial structure varies. For example, mitochondria in skeletal muscle are ovoid structures, with two possible populations: one positioned close to the sarcolemma and the other embedded among the myofibrils [33]. In unstressed or intact nondividing cells, mitochondria exist not as a separate, individual mitochondrion, which is routinely seen in isolated mitochondria as a fractional artifact, but rather as a highly connected reticular network. This reticular network of mitochondria is influenced by fission and fusion executed by mitochondria-shaping proteins called mitodynamins [34]. Fission can be initiated through the response of mitodynamins to mitochondrial energetics, oxidative stress, hypoxia, or mtDNA damage [34]. When a daughter mitochondrion depolarizes following a fission event and is unable to fuse to the reticular network, this solitary mitochondrion will be removed by mitophagy [36]. Structurally, mitochondria exhibit a double-membrane arrangement, which separates the organelle into four distinct compartments (Figure 2): the outer membrane, the intermembrane space, the inner membrane, and the matrix [33, 37]. The outer membrane separates mitochondria from the cytoplasm and surrounds the inner membrane, which separates the intermembrane space from the protein-dense central matrix. Unlike the outer membrane, the inner membrane constitutes a tight diffusion barrier against all ions and molecules and is differentiated into the inner boundary membrane and the cristae, which is the site of mtDNA replication, transcription, protein biosynthesis, and numerous enzymatic reactions. The two regions are continuous at cristae junctions, with cristae extending into the matrix and acting as the primary sites of mitochondrial energy conversion by ATP synthase located in cristae membranes [37]. Mitochondria do not float freely in the cytosol but are positioned in the cytosol with the aid of intermediate filaments and likely through molecular linkages, networks, and bidirectional signaling between xellular components and intermediate filaments [7, 8]. Mitochondrial dynamics are responsible for intracellular distribution and reactions based on functional requirements that are maintained through fission, fusion, growth, and structural reorganization, followed by turnover and rearrangements of mitochondrial proteins and DNA [33, 38–40]. Nucleoid foci containing mtDNA are attached to the cytoskeleton [7] and organize the translation machinery on both sides of the mitochondrial membranes [7]. In view of the organization of general mitochondrial functions (Figure 2), many processes are organized in higher-ordered assemblies, including the respiratory chain supercomplexes [41], endoplasmic reticulum- (ER-) mitochondria complexes [42, 43] involved in organelle biogenesis and inheritance, mitochondrial-contact sites and cristae-organizing-system complexes [44] responsible for the organization of mitochondrial ultrastructure and biogenesis, and mitochondrial membrane supercomplexes that mediate protein trafficking [45, 46]. The detailed description of these complexes is under investigation and is beyond the scope of this review.

## 3. Mitochondrial DNA

Human mtDNA consists of circular, double-stranded 16,569 bp DNA with a contour length of ~5 *μ*m [47], thus requiring mtDNA to be highly packaged to fit into a ~100 nm (in diameter) nucleoid [37]. A mitochondrion contains at least 800 to 1500 proteins of varying relative abundance between tissues [48]; however, mtDNA contains only 37 genes [49, 50] encoding 13 proteins of the mitochondrial respiratory chain, two ribosomal RNAs (12S and 16S), and 22 transfer RNAs (Figure 1). The remaining protein subunits that comprise the OXPHOS, together with those required for mtDNA maintenance, are encoded by nuclear DNA, synthesized by cytoplasmic ribosomes, and are specifically targeted and sorted into their correct locations within the mitochondrion. Unlike nuclear DNA, mtDNA is characterized by high gene density and the absence of introns [51]. With the exception of a ~1 kb noncoding regulatory fragment (D-loop), mtDNA is entirely transcribed as large polycistrons from both strands [51]. Technically, mtDNA in the nucleoid can be localized in fixed cells in two ways: immunolabeling using an anti-DNA antibody or cell growth for one generation in bromodeoxyuridine (BrdU) to uniformly label the DNA to enable detection using an anti-BrdU antibody [7]. Additionally, mtDNA can be stained with 4′,6-diamidino-2-phenylindole, ethidium bromide, or PicoGreen dye [52]. Despite mtDNA being essential for normal physiological functions, the genome is vulnerable to oxidative stress [53]. When isolated rat cardiomyocytes were treated with 50 *μ*M H_2_O_2_, the amount of intact 16 kbp mtDNA decreased by 50% over 10 min, resulting in oxidative stress and leading to mitochondrial dysfunction due to the decline in the activities of complexes I, III, and IV, all of which contain mtDNA-encoded subunits [54]. mtDNA constantly undergoes mutation, with clonal expansion or loss of either point mutations or deletions [12], and mutations of mtDNA, both point mutations and deletions, cause a host of tissue-specific [15] and systemic diseases [12, 55]. The polyploid nature of the mitochondrial genome (up to several thousand copies per cell) gives rise to the important features of homoplasmy, heteroplasmy [56–59], and clonal expansion of mtDNA, even in the same mitochondrion, with random mitochondrial segregations capable of occurring in mitochondria within the same cell [11, 12]. Based on the presence of heteroplasmy and clonal segregation, mtDNA status, regardless of harbored mutations, may not be an important factor in the construction and maintenance of nucleoid organization, given that nucleoid-containing mutated mtDNA can segregate in the cell. Although additional study is required to understand the behavior of nucleoid-containing mutated mtDNA and its propagation, why and how mitochondria (or cells) tolerate this aberrant condition remain an interesting question.

## 4. Mitochondrial Nucleoid Structure and Dynamics

The term nucleoid was first used in 1937 by Piekarski to describe the envelope-lacking structure of the bacterial chromosome as being distinct from that of eukaryotes [60]. Similar DNA-containing structures were later discovered in mitochondria and plastids [61]. Nucleoids do not contain membranes capable of separating the nucleoid compartment from the matrix [27]. The mitochondrial nucleoid is composed of mtDNA and numerous nucleoid-associated proteins (Figure 2) that form a macromolecular structure capable of providing submitochondrial organization of mtDNA [29, 62]. Efficient maintenance of mtDNA in discrete, segregated units located at intervals throughout the mitochondrial network is concerted through the control of nucleoid structure [32].

**Figure 3:**
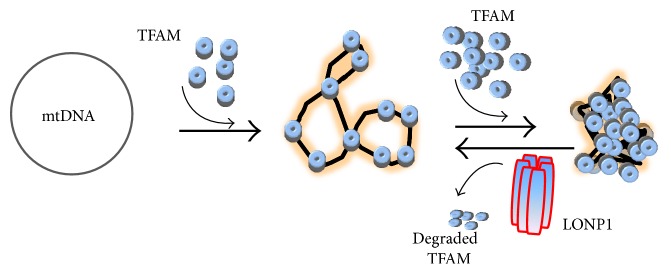
mtDNA packaging by TFAM and its degradation by the mitochondrial AAA protease LONP1. Human circular mtDNA is packaged by TFAMs, but their excessive packing of mtDNA may result in shutdown of mtDNA transcription and replication. TFAM can be degraded by the mitochondrial protease LONP1. LonP1, mitochondrial AAA protease; mtDNA, mitochondrial DNA; TFAM, mitochondrial transcription factor A.

**Figure 4:**
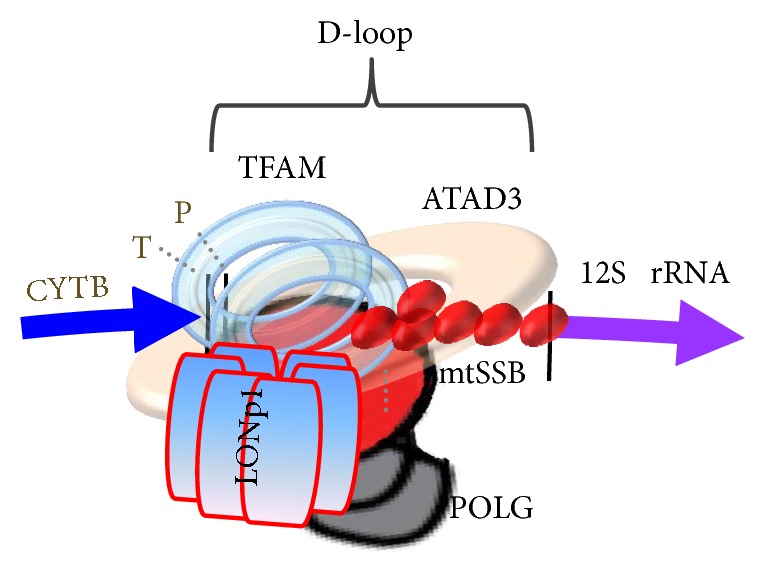
Putative nucleoid-associated proteins located in the D-loop region of mtDNA. TFAM, POLG, ATAD3, LONP1, and mtSSB are nucleoid-associated proteins that possibly interact with the D-loop region of mtDNA. These five proteins might exhibit DNA-binding capacity and, therefore, directly associate with mtDNA. ATAD3, ATPase family AAA-domain-containing protein 3; LONP1, mitochondrial AAA protease; mtDNA, mitochondrial DNA; mtSSB mitochondrial single-stranded DNA-binding protein; POLG, mitochondrial polymerase *γ*; TAFM, mitochondrial transcription factor A.

The organization of the nucleoid is a very fundamental question in mitochondrial biology. The crucial structural difference between nuclear chromatin and mitochondrial nucleoid is that mtDNA is not associated with histones in the form of nucleosomes [20, 63]. Nucleoids are roughly spherical, with a diameter of ~100 nm and with each containing more than one copy of mtDNA [37]. In view of their size, nucleoids must fit into the ~10 *μ*m tubules of the mitochondrial tubular network, which can be approximated by infinite cylinders of ~250 nm to ~400 nm diameter [64, 65]. In human cells, the multilayer model of mitochondrial nucleoid organization [66], which describes separation into the inner core region where DNA and proteins (DNA-packaging proteins and proteins involved in replication and transcription) are concentrated and the outer peripheral region containing proteins temporarily recruited to execute special functions in the nucleoid, was suggested based on the tightly bound mtDNA [35, 61]. mtDNA replication and transcription occur in the core region through the activity of mitochondrial transcription factor A (TFAM), mitochondrial RNA polymerase (POLRMT), mitochondrial single-stranded DNA-binding protein (mtSSB), mitochondrial polymerase *γ* (POLG), and Twinkle helicase, with subsequent RNA processing and translation occurring in the outer region (peripheral region) [22, 61, 67]. In the peripheral region of nucleoids, numerous putative proteins were also identified [61]; however, less is known concerning nucleoid states during mtDNA replication and/or transcription. As shown in Figure 3, mtDNA can be compactly packaged by the binding of mitochondrial transcription factor A (TFAM). The D-loop region of mtDNA, which constitutes a regulatory site for mtDNA replication and transcription, is anchored to the inner mitochondrial membrane (Figures 2 and 3) likely through a multiprotein complex [41, 42, 44] and serves as a central hub for forming nucleoids. As depicted in Figure 4, POLG, ATPase family AAA-domain-containing protein 3 (ATAD3), and the mitochondrial AAA proteases Lon peptidase 1 (LONP1) and mtSSB, including TFAM, are believed to be nucleoid-associated proteins that might interact with the D-loop region of mtDNA [22, 62, 68]. These mtDNA-binding proteins are involved in interactions between mtDNA and the mitochondrial inner membrane, ribosome, and other supercomplexes to facilitate transport of proteins or molecules [45, 69–71]. Mutations in the D-loop region might result in altered binding affinities for the nuclear proteins involved in mtDNA replication and transcription, resulting in severe depletion of mtDNA content due to replication failure and disruption of nucleoid structure [12, 32, 72–75].

## 5. Nucleoid Structure and Dynamics

**Figure 5:**
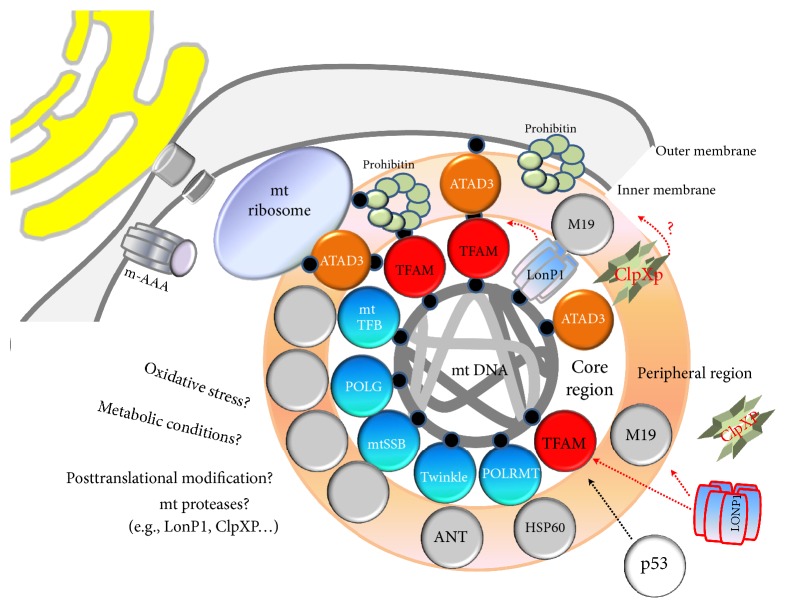
Spatial organization of nucleoid-associated proteins. Some proteins that exhibit mtDNA-binding capacity are located in the core region, whereas others might interact with proteins in the core region or other mitochondrial proteins. ANT, adenine nucleotide translocator; ATAD3, ATPase family AAA-domain-containing protein 3; HSP60, heat-shock protein 60; LONP1, mitochondrial AAA protease; mAAA, mitochondrial ATPase-associated with various cellular activities (AAA+) protease; mtDNA; mitochondrial DNA; mtSSB, mitochondrial single-stranded DNA-binding protein; mTERF, mitochondrial transcription termination factor; PHB; prohibitin; POLG, mitochondrial polymerase *γ*; POLRMT, mitochondrial RNA polymerase; TFB1M, mitochondrial transcription factor B1; TFB2M, mitochondrial transcription factor B2.

Nucleoid structure may vary between tissue types and according to age [76]. Nucleoids are tethered directly or indirectly via the mitochondrial membrane to kinesin and microtubules in the surrounding cytoplasm [7]. Additionally, nucleoids are composed of thin filaments that protrude outward and serve as anchors to the membrane [21]. Movement of nucleoids located in the protein-dense matrix compartment is limited due to their attachment to the mitochondrial inner membrane (Figure 5), which precludes free diffusion through the matrix compartment [7]. A subset of nucleoids can be observed in close proximity to microtubules, which are used to transport mitochondria over long distances and suggest important roles for the cytoskeleton in nucleoid movement, division, and/or sorting [63]. Nucleoids containing nascent mtDNA localize to mitochondrial tips, with these products of division preferentially distributed within cells as compared with nonreplicative nucleoids [70]. In addition, nucleoids actively engaged in mtDNA synthesis in mammalian cells are spatially and temporally linked to a small subset of ER-mitochondria contacts destined for mitochondrial division [37].

During mtDNA transcription or replication, numerous nucleoid-associated proteins are recruited, indicating that the mitochondrial nucleoid is dynamic and not a single discrete entity [7, 77]. The kinetics of replication and transcription (monitored by immunolabeling after incorporation of BrdU or bromouridine) suggest that each mtDNA replicates independently of others and that newly made RNA remains (resident half-life: ~43 min) long after it has been made [7]. During transcription or replication, nucleoids should be relaxed to facilitate attachment of transcription factors; therefore, the size of such active nucleoids might be larger than that of quiescent nucleoids due to the surrounding shell of proteins associated with the replication and transcription machinery [64]. Unlike a single nucleoid, nucleoid clusters will be formed and mostly contain nucleoids surrounding newly replicated mtDNA; however, the nucleoid population not in replication mode remains outside of these clusters [78]. It was suggested that mitochondrial nucleoids can be reversibly clustered with the aid of TFAM upon mtDNA stress and that this nucleoid clustering might be beneficial for newly replicated mtDNA against intercalators, such as the mtDNA-depletion agent ethidium bromide or anticancer drugs [78].

Within the mitochondrion, nucleoids show an asymmetric intracellular distribution determined by mitochondrial division, fusion, and motility events [70], suggesting that the nucleoid-transmission process is DNA-independent and reliant upon protein-protein interactions [63]. At division sites, mtDNA replication occurs upstream of mitochondrial constriction and assembly of the division machinery. In the absence of mtDNA leading to defects in respiratory activity and energy production, nucleoid integrity is lost due to the absence of protein-DNA and additional protein-protein interactions, and the mitochondrial reticulum is compromised due to the reduced cristal-membrane content [79]. However, nucleoid proteins are capable of binding to their proper sites on the inner mitochondrial membrane and are sorted normally in the absence of mtDNA, given the near-uniform distribution of mtSSB in mtDNA-depleted rho-zero cells [80]. The maintenance or selective degradation of mitochondrial nucleoids free of mtDNA remains unknown.

In mammalian cells, mtDNA exhibits a closed, circular form; however, upon strand breakage or partial deletion, this circular structure is linearized. This linearized and unsealed form of mtDNA renders maintenance of intact nucleoid structure difficult due to hindered spatial organization between the mtDNA and nucleoid-constituting proteins. Eventually, nucleoids encapsulating linearized mtDNA may collapse or be degraded by mitochondrial proteases or nucleases. This was reported following application of mitochondria-targeted obligate heterodimeric zinc finger nucleases capable of specifically eliminating pathogenic human mtDNA, thereby altering the ratio of desired haplotypes [81]. Additionally, the presence of deletion mutations in mtDNA present in human mitochondrial diseases [12, 15] suggests that mtDNA size is not a critical factor in nucleoid formation and maintenance; however, additional studies are needed to determine whether the state of mtDNA in either circular or linear form is important in nucleoid construction and maintenance.

Mitochondria are too large and filamentous to achieve spatial uniformity by free diffusion [82]; therefore, mitochondrial nucleoids use active control to ensure their segregation in proportion to the cytoplasmic volume and spacing in semiregular arrays, unlike allocations of chromosomes during cell division [83, 84]. Mitochondrial fission and fusion machinery operate on the two lipid bilayers that surround mitochondria to ensure the efficient distribution of mtDNA throughout the cell and simultaneously protect the nucleoid from fission events at the nucleoid itself [7]. It is likely that the processes of mitochondrial fission, which appears uncoupled to mtDNA replication in nucleoids and occurs adjacent to mtDNA [7, 25] and mtDNA-nucleoid organization, are coordinated to maintain mitochondrial function [83]. During the fission process, nucleoid-partitioning errors are suppressed by controls at two levels: mitochondrial volume is actively distributed throughout a cell and nucleoids are spaced in semiregular arrays within mitochondria. The fusion process is coordinated by mitofusin proteins and optic atrophy 1 (OPA1), guanosine triphosphatases located at the outer mitochondrial membrane [9, 33]. OPA1 generates a peptide, including the exon-4b domain associated with the inner membrane and crucial for mtDNA maintenance that directly interacts with nucleoids and allows their distribution within the mitochondrial network to promote mitochondrial genome replication [85, 86]. It remains unclear what nucleoid-associated factors are intricately connected to the fission and fusion machinery to ensure nucleoid propagation. From an elegant experiment using fluorescent-protein constructs and fusion-protein expression in HepG2 cells, it was speculated that nucleoid redistribution could occur following fission and its subsequent reintegration into the mitochondrial network [65]. However, due to the highly dynamic state of mitochondria and the existing hurdles in visualizing mitochondrial nucleoids in intact cells [65], our understating of nucleoid distribution within the mitochondrial network upon network-morphological changes remains limited. Apart from nucleoid redistribution in the cell, it remains unclear whether the nucleoid fission/reintegration process is involved in compensating for or selectively segregating nucleoids containing mutated mtDNA.

## 6. mtDNA Content in the Mitochondrial Nucleoid

In mitochondrial genetics, the number of mtDNA(s) contained within a single nucleoid is a fundamental question that remains somewhat controversial. Strong discrepancies in mtDNA number present in a single nucleoid might be associated with methodological differences, different cell types, or the unveiled complex behaviors of a nucleoid [22, 87]. According to stimulated emission-depletion microscopy or photoactivated light microscopy [22], mammalian cells might contain an average of 1.45 mtDNA molecules per nucleoid (ranging from ~2.4 to ~7.8 per nucleoid). However, a recent study of mitochondrial nucleoids from mouse embryonic fibroblasts reported that a single nucleoid could contain more than two mtDNA molecules based on the characteristics of TFAM-mediated mtDNA packaging indicating a spherical shape [87]. These different points regarding mtDNA-molecule population within a single nucleoid highlight the continued importance of understanding nucleoid ultrastructure, but questions concerning control of individual mtDNA transcription and replication nucleoids remain unsolved. Interestingly, mtDNA does not mix between two different nucleoids, despite their proximity in space and time within the mitochondrial network, but rather, mitochondrial nucleoids are tightly regulated by their genetic content rather than the free exchange of mtDNAs [88]. However, heterologous mtDNAs within maximal diffusible distance of mtDNA transcripts in the same mitochondrion can transcomplement to restore mitochondrial function, a result that provides a basis for future research in mitochondrial therapeutics [32, 83].

## 7. Mitochondrial Nucleoid-Associating Proteins

In the previous section, the characteristics of mitochondrial nucleoids were briefly addressed (Figures 1–5). Principally, nucleoid-associated proteins can be defined as any protein that either temporarily or permanently associates directly with mtDNA or with other nucleoid proteins and plays roles in mtDNA maintenance [28]. To better understand the biological functions and regulation of mitochondrial nucleoids, identification of proteins involved in nucleoid formation is necessary [89]. Except for conserved TFAM and mtSSB [19, 53], there is no consensus regarding nucleoid composition due to differences in cell types or tissues used for preparations, the various biochemical approaches used for examination based on noncovalent protein-DNA and protein-protein interactions [90], formaldehyde cross-linking [91] or proximity-based biotinylation techniques [43], the low abundance of proteins within mitochondrial nucleoids, and the limited characterization of proteins related to mtDNA maintenance and gene expression [89]. Additionally, it is difficult to use genetic methods to study these associations, because all of the proteins identified are likely required to maintain mitochondrial function [67].

Nucleoids from most organisms contain ≥50 proteins, many of which have not been characterized with respect to nucleoid function [22, 28, 66, 67, 92–94]. Generally, proteins involved in mtDNA packaging or covering exhibit low molecular weight and function as multimers (Table 1). Nucleoid-associated proteins from various organisms can be classified into at least four groups: (1) proteins with known functions in DNA transactions and packaging, (2) proteins participating in protein quality control, (3) bifunctional metabolic enzymes with various activities, and (4) cytoskeletal components [63]. Some examples of these nucleoid-associated proteins are presented in Table 1 and Figure 5. Among those identified as nucleoid-associated proteins, many exhibit identifiable activities unrelated directly to mtDNA maintenance, suggesting that their bifunctionality might involve participation in both mitochondrial metabolism and mtDNA maintenance [32, 89, 95]. Interestingly, mutations in proteins associated with the mitochondrial nucleoid might cause either the loss of mtDNA content from the cell or generation of mtDNA mutations [32, 96].

### 7.1. Mitochondrial TFAM

TFAM is a nuclear-DNA-encoded 24-kDa protein containing two high-mobility group- (HMG-) box domains and able to bend, wrap, and unwind DNA through modes involving sliding, collisions, and patch formation [29, 89, 90, 97–101]. TFAMs cover mtDNA with a footprint of between 10 bp and 30 bp (Figure 3()) and mediate the tight compaction of relaxed mtDNA [62]. In addition to a role as a master transcription factor, TFAM plays an equally important role in promoter selection, initiation of genome replication, and the regulation of mtDNA copy number [98]. TFAM concentration can increase mtDNA content through its preferential binding at the light-strand promoter (LSP) in the D-loop and TFAM-mediated stabilization of mtDNA, perhaps by reducing the rate of DNA turnover [98]. The molecular ratio of TFAM relative to mtDNA is ~900 : 1 in human mitochondria [102], with this amount of TFAM sufficient to coat 16.6 kbp circular human mtDNA [96]. Theoretically, mtDNA density, TFAM/mtDNA stoichiometry, or TFAM density within a single nucleoid may differ under various physiological (mtDNA transcription or replication) or pathological conditions [64], thereby implying the presence of mechanisms that select only a subset of mtDNA molecules for replication, with others remaining in a silent state [101]. A recent finding suggested that human TFAM plays an important role in the equal distribution and symmetric segregation of mtDNA in cultured cells [66]. For example, enlarged mtDNA nucleoids have been observed in both TFAM-knockdown HeLa cells and TFAM-overexpressing mice [103]. The process of uncoating mtDNA has not been elucidated but likely involves the selective and processive dissociation of TFAM [104]. In view of TFAM turnover [7], LONP1 determines the proteolytic degradation of TFAM and constitutes an additional step in controlling mtDNA content (Figure 3()). Apart from regulating TFAM expression and turnover, posttranslation modification of TFAM by glycosylation, phosphorylation, acetylation, or ubiquitination might constitute alternative control points of TFAM activity, given that these modifications can influence DNA-binding activity, protein-protein interactions, homodimerization, or cooperative-binding characteristics [98]. For example, TFAM can be phosphorylated within its HMG1 domain by cyclic adenosine monophosphate-dependent protein kinase in mitochondria, thereby impairing its ability to bind DNA and activate transcription [104]. By contrast, in the cytosol, TFAM phosphorylation might alter its degradation by the proteasome or its association with mitochondrial protein-translocation machinery [104].

### 7.2. Mitochondrial Transcription and Replication Machinery

The minimal proteins required for mtDNA transcription (e.g., POLRMT and mitochondrial transcription factor B (mtTFB)) and replication (POLG and POLG2) are embedded in the core region of nucleoids through their mtDNA-binding capabilities [13, 43, 62]. Due to the high degree of compaction in mtDNA, mitochondrial topoisomerase 1, found in the core region of mitochondrial nucleoids, is required during replication to ease torsional strain resulting from replication progression [62]. Additionally, POLRMT and mtTFB, located in the core region of nucleoids, are necessary for mitochondrial transcription [13, 62].

### 7.3. mtSSB

mtSSB is a 16-kDa protein that forms a tetramer and binds ssDNA with high affinity in a sequence-independent manner, thereby aiding DNA replication, recombination, and repair processes [64, 105]. Similar to TFAM, mtSSB is a major nucleoid-associated protein also involved in mtDNA/nucleoid distribution within the mitochondrial network [106]. Additionally, mtSSB influences mitochondrial biogenesis [105, 106], and its downregulation leads to increases in morphological alterations, such as fragmentation or elongation, of mitochondria [107].

### 7.4. Twinkle Helicase

Twinkle helicase is a nucleoid-associated protein found in the core region [66] and the only known mitochondrial helicase involved in unwinding mtDNA during the replication process, synthesis of the nascent D-loop strand, and completion of mtDNA replication [108, 109]. Decreases in Twinkle helicase concentration result in mtDNA depletion, whereas overexpression leads to increases in mtDNA copy number [109]. Many disease-causing mutations, including autosomal dominant progressive external opthalmoplegia, have been mapped to the Twinkle helicase gene, with mutation resulting in defects in OXPHOS and the onset of neuromuscular symptoms [109]. Twinkle helicase might also promote nucleoid attachment to membrane structures highly enriched in cholesterol, thereby providing a replication platform at ER-mitochondrial junctions [71].

### 7.5. Mitochondrial ATPase AAA-Domain-Containing Proteins (AAA)

Several ATP-dependent proteases, including LONP1, ATP-dependent Clp protease ATP-binding subunit ClpX-like protein, and m-AAA protease, localize to the mitochondrial matrix [96] and are associated with the peripheral region of nucleoids [61, 96, 110]. Among these, LONP1 is a quality control enzyme that degrades oxidatively modified and misfolded proteins and also binds to specific regions of the mitochondrial genome, including ssDNA in both the LSP region and RNA produced from the LSP region [98]. Additionally, LONP1 might recognize oxidized TFAM or degrade unbound TFAM; alternatively, LONP1 can also remove TFAM from oxidatively modified DNA, to which TFAM binds with higher efficiency than it does unmodified DNA [98]. Although the triggers for LONP1-mediated TFAM degradation remain unclear, mitochondrial stress might activate LONP1 activity to initiate TFAM degradation and activate transcription in quiescent mtDNAs [98]. Interestingly, LONP1 expression decreases with age or exposure to chronic stress, possibly resulting in accumulation of oxidized proteins and disturbance of the nucleoid dynamics [111].

### 7.6. ATAD3

ATAD3A and the less abundant ATAD3B are protein paralogs that form heterohexamers or homohexamers with ATAD3A and extend from the inner membrane into the outer mitochondrial membrane [66, 112]. ATAD3 was discovered as an important membrane-bound mitochondrial ATPase [112]. Although ATAD3 appeared to be bound to the D-loop of mtDNA in nucleoid [75], subsequent experiments indicated that ATAD3 made direct contact with mtDNA but is among the nucleoid-associated proteins involved in connections between mitochondrial nucleoids and mitochondrial ribosomes [66, 93]. ATAD3 associates with ER-mitochondrial junctions and holds together Twinkle helicase-containing mammalian nucleoids attached to membrane structures highly enriched in cholesterol [71]. Therefore, ATAD3 also plays an important role in nucleoid positioning in human mitochondria, with altered ATAD3 expression disturbing mtDNA maintenance and replication [77].

### 7.7. Prohibitin

Prohibitin proteins (PHB1 and PHB2) are membrane-anchored molecular chaperones and protein stabilizers [67, 103]. In addition to pleiotropic functions, including apoptosis, in mitochondria, PHB1 is required for the organization and stability of mitochondrial nucleoids either through a TFAM-dependent or through a TFAM-independent pathway, in which it regulates nucleoid organization directly or through undefined nucleoid factors [113]. Several reports supported the notion that PHBs are important in mtDNA copy number regulation [103, 113].

### 7.8. Other Putative Nucleoid-Associated Proteins

A group of heat-shock proteins (HSPs) are associated with nucleoids in both yeast and human cells [63]. HSP60 functions both in mitochondrial protein import and as a nucleoid protein required for nucleoid division [63]. The components of detergent-resistant mtDNA nucleoids include adenine nucleotide translocator (ANT), the E2 subunits of two large dehydrogenase complexes, pyruvate dehydrogenase, and branched-chain keto acid dehydrogenase without association with other subunits [67].

## 8. Links between Mitochondrial Nucleoid Composition and Metabolic Control

Mitochondrial nucleoids undergo remodeling, such as transition of its structure or recruitment of other proteins that influence nucleoid-related activities in response to metabolic cues in yeast [114, 115]. There is less concrete evidence of yeast-like nucleoid remodeling in mammalian systems; however, nucleoid remodeling might be possible according to metabolic demand [5, 32], because access to mtDNA for transcription, translation, and replication is highly coordinated by various factors inside and outside of the nucleoid compartment. Moreover, nucleoid-associated proteins are directly involved in not only mtDNA maintenance and propagation but also metabolic activities not directly linked to mtDNA stability [62]. Additionally, retrograde signaling from the mitochondria to the nucleus can be promoted through interactions between mtDNA and nucleoid-associated factors [2, 7]. It is unclear whether nucleoid-associated proteins can directly regulate mitochondrial gene expression or bioenergetics.

## 9. Pathological Changes Associated with Mitochondrial Nucleoids

Mitochondrial morphology is coupled to function, as a loss in mitochondrial bioenergetic capacity results in an inability to maintain a highly ordered structure [62]. The shaping, maintenance, and dissociation of nucleoids in a mitochondrion is undertaken by numerous proteins that communicate with one another and the nucleoid in order to determine cellular demands dependent upon physiological conditions. The general principles of nucleoid organization and its pathological implications remain unclear; however, significant interest in the role of nucleoids and their impact on mitochondria-related diseases has focused on their association with the accumulation of numerous mtDNA mutations [27]. Mutations in mtDNA and/or aberrant nucleoid organization might be a causal factor in etiologies of various diseases, including cancers [15, 106]. In addition to mutations or damage to nucleoid-associated proteins, aberrant interactions between or dysfunction of nucleoid-interacting proteins causes pathological conditions due to failed mtDNA maintenance. For example, ANT1 interacts with mtDNA [67], and its mutation causes a genetic disorder leading to multiple mtDNA deletions and autosomal dominant progressive external opthalmoplegia [116]. Additionally, the subunits of complex I and the E2 subunits of ATP synthase and 2-oxo-acid dehydrogenase have been identified in nucleoids and are involved in mitochondrial diseases and aging [67]. Under physiological or various cellular stress conditions, p53 can maintain nuclear genome stability through the repair of damaged DNA and the integration of cell-death-signaling pathways with DNA-damage checkpoints [117]. Recently, an additional role for p53 as guardian of the mitochondrial genome was suggested [118]. Mitochondria-translocated p53 can interact with TFAM and POLG located in the core region of nucleoids and involved in mtDNA maintenance [119]. It was suggested that the expression of dynamin-related protein 1 and OPA1 involved in mitochondrial dynamics is regulated by p53 [118]. Interestingly, human mtDNA also contains a putative p53-binding sequence [120], suggesting that p53 functions involve both the nuclear and mitochondrial subcellular compartments and are responsible for maintaining mtDNA integrity through its activities in both regions (Figure 5). It remains unclear whether p53 directly affects the structure and dynamics of mitochondrial nucleoids.

Oxidative damage can disturb the regulation of nucleoid dynamics. For example, oxidized mtDNA is degraded by lysosomes; however, oxidized mitochondrial nucleoids are not degraded via the lysosomal pathway in neutrophils in human lupus, resulting in activation of type I interferon production [121]. Oxidative stress may deteriorate the dynamics of nucleoids due to their resulting structural modifications and the breakdown of redox control, resulting in mitochondrial dysfunction. However, more extensive work is needed to clarify the mechanisms associated with oxidative-stress-mediated disruption and/or dysfunction of mitochondrial nucleoids. The clustering of multiple mtDNA genomes into a single nucleoid complex might promote the progressive age-related accumulation of deletions and point mutations in mtDNA in many somatic tissues and particularly in postmitotic cells. By contrast, oocytes appear to have the ability to select against deleterious mutations in mtDNA, at least in mice [17]. Therefore, the processes by which nucleoids are actively chosen for mtDNA replication and distribution within mitochondrial networks are not clearly understood and remain as highly relevant issues associated with understanding the basis of human metabolic diseases, aging, and neurodegenerative disorders caused by mtDNA mutations, as well as those in nuclear genes, that affect mtDNA maintenance [70].

## 10. Concluding Remarks and Perspectives

In mitochondrial biology and its role in human diseases, nucleoids remain an unexplored feature. Their role as entities that organize mtDNA by forming complexes with accessory proteins, as well as regulators of gene expression, greatly influences the phenotypic expression of mtDNA defects [17]. In this review, we addressed the newly emerging field of nucleoid research, including investigations of its structure and dynamic regulation. Nucleoid-associated proteins function as building blocks of nucleoids, which are intimately involved in mitochondrial genetics and the fine tuning of metabolic demands (Figure 5). To understand the complex behavior of nucleoids, it will be necessary to examine the specific interactions between different nucleoid-associated proteins and mtDNA to definitively elucidate their roles in nucleoid organization. In addition to posttranslational modifications of nucleoid-associated proteins, oxidative changes that occur in nucleoid-associated proteins and their impact on mtDNA likely influence nucleoid dynamics and function and might be necessary to understand the real functional role of nucleoid and mitochondria. Furthermore, the assembly and dynamic control of nucleoid structure involving mtDNA also remains unclear and should be the subject of future investigation.

Collectively, mtDNA is preserved in a highly ordered manner by nucleoids. Mitochondrial nucleoids act not as simple shields or parcels for mtDNA but constitute a switch for controlling mitochondrial metabolism in response to cellular demands. New findings associated with mitochondria should be interpreted in conjunction with nucleoid dynamics to fully understand its overall physiological and pathophysiological role.

## Figures and Tables

**Table 1:** Mitochondrial nucleoid-associated proteins with known functions.

Nucleoid protein	Location/shape	Function in mtDNA metabolism and/or nucleoid	Reference
Mitochondrial transcription factor A (TFAM)	Core region/homodimerization	(i) Transcription initiation	[66, 89, 90]
		(ii) mtDNA binding, bending, and packaging	
		(iii) mtDNA copy number regulation and segregation	
Mitochondrial single-stranded DNA-binding protein (mtSSB)	Core region	(i) Single-stranded mtDNA binding	[66, 89, 90, 122]
		(ii) Coating of single strands of mtDNA	
ATP-dependent Lon protease (LONP1)	Core region/homo-oligomeric ring	(i) Binding to G-rich single-stranded mtDNA	[63, 66, 96, 123]
		(ii) Interaction with POLG and Twinkle	
		(iii) mtDNA quality control	
		(iv) TFAM degradation	
Twinkle	Core region/hexamer	(i) mtDNA replication	[66]
mtDNA polymerase *γ* (POLG/POLG2)	Core region	(i) mtDNA replication and repair	[66, 89, 90, 122]
Mitochondrial transcription factor B1/B2 (TFB2M/TFB1M)	Core region	(i) Transcription	[66]
Mitochondrial RNA polymerase (POLRMT)	Core region	(i) Transcription	[66]
Mitochondrial transcription termination factor (mTERF)	Core region	(i) Transcription and replication	[94]
Mitochondrial topoisomerase I (TOP1M)	Core region	(i) Replication	[62]
ATPase AAA-domain-containing protein 3 (ATAD3)	Peripheral region/hexamer	(i) Binding to the D-loop region of mtDNA	[62, 66]
		(ii) Molecular scaffolding for mitochondrial translation via association with both the mitochondrial inner membrane and the ribosome	
		(iii) Organization and segregation of nucleoids	
Prohibitins 1 and 2 (PHB1 and PHB2)	Peripheral region/oligomeric ring	(i) Stabilizing TFAM in mitochondrial nucleoids	[62]
		(ii) Maintenance of mitochondrial morphology	
		(iii) Regulation of mtDNA copy number	
Mitochondrial nucleoid factor 1 (MNF1 or M19)	Peripheral zone	(i) Linking between mtDNA and mitochondrial ATP production	[76]
		(ii) mtDNA translation	
		(iii) Assembly of respiratory complexes	
Mitochondrial AAA protein ClpX	Peripheral region (?)	(i) mtDNA distribution	[66]
		(ii) TFAM quality control	
Leucine-rich pentatricopeptide-repeat motif-containing protein 130 (LRP130)	Peripheral region (?)	(i) Increased mtDNA transcription and RNA stability	[124]
